# Differential Synovial CGRP/RAMP1 Expression in Men and Women With Knee Osteoarthritis

**DOI:** 10.7759/cureus.15483

**Published:** 2021-06-06

**Authors:** Kentaro Uchida, Shotaro Takano, Ken Takata, Manabu Mukai, Tomohisa Koyama, Yoshihisa Ohashi, Hiroki Saito, Masashi Takaso, Masayuki Miyagi, Gen Inoue

**Affiliations:** 1 Department of Orthopaedic Surgery, Kitasato University, School of Medicine, Sagamihara, JPN; 2 Department of Orthopaedic Surgery, Kitasato University School of Medicine, Sagamihara, JPN; 3 Department of Orthopedic Surgery, Kitasato University School of Medicine, Sagamihara, JPN; 4 Orthopaedic Surgery, Kitasato University, Sagamihara, JPN

**Keywords:** calcitonin gene-related peptide, receptor activity modifying protein 1, osteoarthritis, pain, synovium

## Abstract

Background

Female patients with osteoarthritis report more severe knee pain compared to men. However, the mechanism underlying sex differences in pain remains unclear. We previously found that calcitonin gene-related peptide (CGRP) was expressed in synovial tissue and that this localization may play a role in pain associated with knee osteoarthritis (KOA). Several animal studies have shown that the expression of CGRP and its receptor (receptor activity modifying protein 1, RAMP1) differs by sex. Here, we investigated synovial CGRP and RAMP1 expression in male and female patients with KOA.

Methods

Synovial tissue (ST) was harvested from male and female subjects (n=30 each) with radiographically confirmed unilateral Kellgren/Lawrence grade 3-4 KOA during total knee arthroplasty. Patients’ subjective pain severity was scored on a 0 to 10 cm visual analog scale (VAS). We compared the expression of CGRP and RAMP1 in ST from men and women and examined the correlation between mRNA levels of CGRP and RAMP1 and pain severity.

Results

Synovial expression of CGRP and RAMP1 was significantly elevated in women compared to men (CGRP, P=0.017; RAMP1, P=0.028). While CGRP expression was positively correlated with pain severity in females (ρ=0.443, P=0.014), no correlation was observed in men (ρ=-0.021, P=0.913). RAMP1 expression was not correlated with pain severity in either men or women (male, ρ=-0.114, P=0.939; female, ρ=-0.047, P=0.807).

Conclusion

CGRP and RAMP1 expression levels differ between men and women. Differential CGRP levels may suggest the presence of different pain mechanisms in men and women with KOA.

## Introduction

Compared to men, women have an elevated risk of developing knee osteoarthritis (KOA) [1, 2]. Additionally, women with osteoarthritis (OA) reported more severe knee pain and reduced function [3, 4]. Several studies have examined sex differences in pain sensitivity in healthy subjects. A meta-analysis identified elevated pain sensitivity in women compared to men based on their pain threshold towards various noxious stimuli [5]. Further, a clinical study demonstrated that women report more severe knee pain compared to men with a similar grade of radiographic KOA [6]. However, the mechanism underlying the difference in pain sensitivity between men and women remains unclear.

Calcitonin gene-related peptide (CGRP), a neuropeptide of 37 amino acids, binds to the calcitonin receptor-like receptor (CLR) and receptor activity-modifying protein 1 (RAMP1) [7]. With vasodilatory effects, CGRP is an important factor in migraine pain formation, with a clinical trial showing that small molecule CGRP receptor antagonists have beneficial effects in the treatment of migraine pain [8]. Additionally, studies have suggested that there may be an important link between synovial CGRP levels and OA-related pain [9, 10]. Interestingly, levels of CGRP and its receptors have been shown to differ by sex in rats and humans [11-16]. We hypothesized that the expression levels of CGRP and its receptors in osteoarthritic joints differ between men and women.

Herein, we investigated the synovial expression of CGRP and RAMP1 in male and female patients with knee OA.

## Materials and methods

Ethics approval and patient consent to participate

Ethics approval was obtained from our institutional review board (IRB) (Approval number: B13-113). Informed consent to participate in this study was obtained from all patients the day prior to surgery, in accordance with the Declaration of Helsinki. Patients were excluded based on the presence of specific comorbid conditions, including the following: metastatic cancer; rheumatoid arthritis, fibromyalgia, or other systemic rheumatic diseases; history of gout in knee or hip (more on this can be found in Material and Methods section of this article).

Samples

We harvested synovial tissue (ST) from the suprapatellar pouch of the operated knee of 60 patients (30 men and 30 women) with KOA during total knee replacement surgery. ST samples were instantly frozen in liquid nitrogen and then stored at −80°C until RNA extraction.

Real-time PCR

Procedures used for RNA extraction, cDNA synthesis, and quantitative RT-PCR (qRT-PCR) were the same as those reported previously [9, 10, 17, 18]. Primers against CGRP, RAMP1, and GAPDH were synthesized based on previous studies (Table 1) [9]. We evaluated levels of CGRP and RAMP1 mRNA in ST by dividing by levels of GAPDH using the ∆∆CT method (delta-delta cycle threshold method). We compared the expression of CGRP and RAMP1 in ST between the men and women in the study and evaluated the relationship between expression levels of CGRP and RAMP1 and pain, as determined using a 1-10 cm visual analog scale (VAS). To evaluate the effect of OA severity on CGRP and RAMP1 expression, we stratified patients based on KOA severity and compared those with Kellgren/Lawrence (KL) grade 3 to those with KL 4.

**Table 1:** Sequences of the primers used in this study

Gene	Direction	Primer Sequence (5¢–3¢)	Product Size (bp)
CGRP	F	TTGCCCAGAAGAGAGCCTGTG	91
	R	TTGTTCTTCACCACACCCCCTG	
RAMP1	F	GGCCTCTGGCTGCTCCTG	172
	R	GCTCCCTGTAGCTCCTGATG	
GAPDH	F	TGTTGCCATCAATGACCCCTT	202
	R	CTCCACGACGTACTCAGCG	

Statistical analysis

All statistical analyses were conducted using SPSS 25.0. Categorical and continuous variables were compared using the chi-squared test and Mann-Whitney U test, respectively. The relationship between CGRP and RAMP1 and pain severity was evaluated using Spearman’s correlation coefficient. Statistical significance was defined as P<0.05.

## Results

Expression of CGRP and RAMP1 in ST from male and female KOA patients

**Figure 1:**
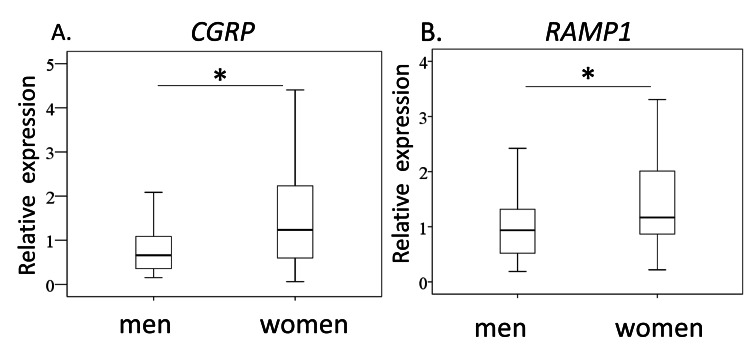
Synovial CGRP and RAMP1 expression men and women with knee osteoarthritis Quantitative RT-PCR analysis for CGRP (A) and RAMP1(B). * P<0.05

We studied CGRP and RAMP1 expression in ST from male and female KOA patients. Age, bone mass index (BMI), K/L grade 3/4 ratio, and VAS score were comparable between sexes (Table 2). In contrast, CGRP and RAMP1 expression were significantly higher in women than in men (CGRP, P=0.017; RAMP1, P=0.028; Figure 1).

**Table 2:** Patients’ demographics BMI, bone mass index; K/L, Kellgren/Lawrence; VAS, visual analog scale

	Male	Female	P-value
Age (years)	72.9±10.5	74.4±6.7	0.824
BMI (kg/m^2^)	27.1±3.2	26.6±4.3	0.258
KL grade (3,4) n	11,19	13,17	0.792
VAS (cm)	6.4±2.5	7.3±2.3	0.149

Relationship between CGRP and RAMP1 expression and pain severity in men and women

**Figure 2:**
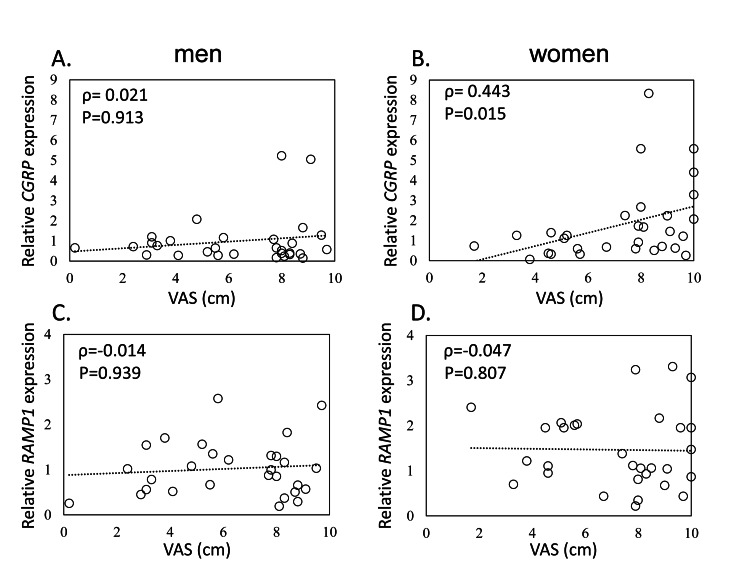
Relationship between CGRP and RAMP1 expression and pain severity in men and women Relationship between visual analog scale (VAS) and CGRP in men (A) and women (B), and RAMP1 in men (C) and women (D)

While CGRP expression was positively correlated with pain severity in women (ρ=0.443, P=0.014), no correlation was observed in men (ρ=-0.021, P=0.913). Further, RAMP1 expression was not correlated with pain severity in men or women (male, ρ=-0.114, P=0.939; female, ρ=-0.047, P=0.807; Figure 2).

Effect of OA severity on CGRP and RAMP1 expression

**Figure 3:**
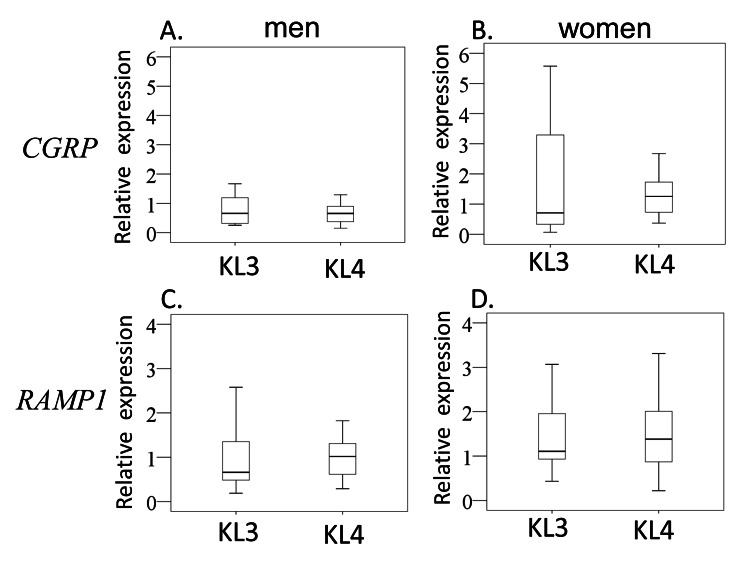
Effect of OA severity on CGRP and RAMP1 expression Effect of OA severity on CGRP expression in men (A) and women (B) and RAMP1 expression in men (C) and women (D).

Age, BMI, and VAS score were comparable between patients with KL3 and KL4 for both men and women (Table 3). Likewise, there was no significant difference in CGRP or RAMP1 expression between patients with KL3 and KL4 for both men and women (Figures 3A-D).

**Table 3:** Comparison of radiographic osteoarthritis severity and demographics between male and female patients BMI, bone mass index; VAS, visual analog scale.

	Male			Female		
	KL3 (n=11)	KL4 (n=19)	P-value	KL3 (n=13)	KL4 (n=17)	P-value
Age (years)	68.9±11.4	75.6±9.1	0.085	72.3±6.7	76.1±6.5	0.333
BMI (kg/m^2^)	28.1±2.6	26.6±3.5	0.149	27.1±4.1	26.3±4.6	0.338
VAS (cm)	5.8±2.9	6.7±2.3	0.438	7.9±2.1	6.8±2.4	0.180

## Discussion

Previous studies have reported that the expression of CGRP and RAMP1 differs by sex and is affected by sex hormone levels in serum and several tissues [11-13, 16, 19]. Women have higher plasma CGRP levels than men [14]. Further, an estrogen deficiency has been shown to increase CGRP levels within the lumbar dorsal root ganglion (DRG), medial preoptic nucleus of the hypothalamus, as well as in the midbrain periaqueductal gray in rats [12, 16]. Meanwhile, estrogen treatment in rats leads to reduced CGRP expression in the DRG and trigeminal nucleus caudalis [13]. In contrast, a previous study showed that ovariectomy significantly raises CGRP levels in the trigeminal ganglia relative to the control rats and that estrogen replacement suppresses this increase [11]. Moreover, treatment of ovariectomized rats with estradiol-17β lowered RAMP1 mRNA expression in uterine tissues [19]. In our study, female KOA patients had higher synovial CGRP and RAMP1 expression levels than male patients. As all the female KOA patients were postmenopausal, we predict that the increase in synovial CGRP and RAMP1 expression may have been caused by estrogen deficiency.

In our previous studies, we found that CGRP was expressed in ST and that this expression was correlated with pain in KOA patients comprising both men and women [9, 10]. In the present study, we found that synovial CGRP expression level was correlated with the VAS pain score in women but not men. A previous study suggested that sensitivity toward CGRP differed between male and female rats, with mesenteric arteries isolated from females showing heightened vasorelaxation sensitivity to CGRP [20]. As RAMP1 expression was also higher in women than men in our study, sensitivity to CGRP may partly explain the correlation between CGRP expression and pain in women but not men.

Two limitations of the present study warrant mention. First, the present study lacked healthy control data. Second, the sample size was small (n=30).

## Conclusions

Female KOA patients had higher CGRP and RAMP1 expression in ST than men, and CGRP expression level correlated with pain severity. Sex differences in the CGRP pathway may provide valuable insight for the development of gender-specific treatments.
